# Investigation of incidence and geographic distribution of gliomas in Canada from 1992 to 2010: a national population-based study highlighting the importance of exposure to airport operations

**DOI:** 10.3389/fonc.2023.1190366

**Published:** 2023-05-16

**Authors:** Xinyu Ji, Akram Alakel, Feras M. Ghazawi, Matthew Tsang, Andrei Zubarev, Oliver J. Lasry, Ivan V. Litvinov

**Affiliations:** ^1^ Department Medicine, Faculty of Medicine and Health Sciences, McGill University, Montreal, QC, Canada; ^2^ Faculty of Dentistry, McGill University, Montreal, QC, Canada; ^3^ Department of Medicine, University of Ottawa, Ottawa, ON, Canada; ^4^ Department of Pathology and Laboratory Medicine, University of Ottawa, Ottawa, ON, Canada; ^5^ Division of Dermatology, McGill University, Montreal, QC, Canada; ^6^ Department of Neurosurgery, McGill University, Montreal, QC, Canada; ^7^ Department of Experimental Medicine, McGill University, Montreal, QC, Canada; ^8^ Cancer Research Program, Research-Institute-McGill University Health Centre, Montreal, QC, Canada

**Keywords:** glioblastoma, epidemiology, public health, airport operations, ultrafine particles (UFPs), glioma

## Abstract

**Background:**

Gliomas account for over two-thirds of all malignant brain tumors and have few established risk factors beyond family history and exposure to ionizing radiation. Importantly, recent studies highlighted the exposure to ultrafine particles (UFP) as a putative risk factor for malignant brain tumors.

**Methods:**

Clinical and geographic data encompassing all provinces and territories from 1992 to 2010 was obtained from the Canadian Cancer Registry and Le Registre Québécois du Cancer. Linear regression and joinpoint analyses were performed to assess incidence trends. Significantly higher and lower incidence postal codes were then interrogated using Standard Industrial Classification codes to detect significant industrial activity.

**Results:**

In Canada, between 1992 and 2010, there were ~32,360 cases of glioma. Of these, 17,115 (52.9%) were glioblastoma. The overall crude incidence rates of 5.45 and 2.87 cases per 100,000 individuals per year for gliomas and glioblastomas, respectively, were identified. Our findings further revealed increasing crude incidence of gliomas/glioblastomas over time. A male predominance was observed. Provinces leading in glioma incidence included Quebec, Nova Scotia, and New Brunswick. Significantly lower crude incidence of glioma was found in Nunavut, Northwest Territories, Ontario, and Alberta. A putative regional clustering of gliomas was observed, with higher incidence rates in postal code areas correlating with industrial activity related to airport operations.

**Conclusion:**

This study describes the geographic distribution of the glioma disease burden and, potentially, identifies industrial activity related to airport operations as potentially being associated with higher incidence of this cancer.

## Introduction

In Canada, cancer is the leading cause of mortality (1). Latest Cancer Statistics data indicate that brain and central nervous system cancers account for 1-2% of all new malignancies (1, 2). Gliomas, which represent over two-thirds of all malignant brain tumors, originate in the glial cells of the central nervous system (CNS) (3). The World Health Organization (WHO) classifies gliomas as astrocytoma, oligodendroglioma, and ependymoma (3). They present with distinctive genetic mutations, prognostic factors, and response to treatment. Many gliomas, particularly higher-grade tumors such as glioblastoma, are highly invasive and are associated with poor survival (3). As such, determining risk factors associated with the development of such tumors and understanding their epidemiologic distribution is necessary in order to develop effective strategies to prevent and treat this disease.

Established risk factors for CNS malignancies include exposure to ionizing radiation, concurrent diagnosis of a hereditary cancer syndrome (4, 5) and, the absence of allergies (6–9). Ionizing radiation is known to cause a multitude of cancers (10), and in the CNS, exposure to high or moderate doses of medical and environmental ionizing radiation is the only well-established external risk factor for various brain tumors (4, 5). However, even low doses of radiation averaging 1.5 Gy for tinea capitis of the scalp were associated with relative risks of 18, 10, and 3 for nerve sheath tumors, meningiomas, and gliomas, respectively (4, 5). Early age of radiation exposure is associated with a higher risk of CNS malignancy later in life and may explain the obstacles in recognizing causal environmental relations in epidemiologic investigations (11). Interestingly, allergic conditions and higher IgE levels are associated with lower tumor incidences and are hypothesized to enhance tumor surveillance and destruction (6, 8, 9).

Notably, exposure to ultrafine particles (UFPs) may be emerging as a novel risk factor for brain cancers (12, 13). Specifically, a Canadian study recently highlighted a possible association of UFPs and risk of malignant brain tumors (12). UFPs are a subtype of particulate matter that are smaller than 100 nm (≤0.1 µm) in diameter that may be absorbed *via* alveoli and carried systemically to the CNS or may cross the blood brain barrier though the nose and olfactory circulation (13). Notably, it was documented that increased levels of UFP may be found in the proximity of airports and may be associated with brain cancers (13). Other suspected associations include environmental pollution, cellphone use, malnutrition, and socio-economic status. However, these risk factors remain to be ascertained (14).

Importantly, up to 5% of grade II gliomas can be asymptomatic (4, 15). Hence, medical imaging likely contributes to the discovery of these tumors. Medical imaging is more likely to occur in metropolitan areas in comparison to remote rural communities.

As highlighted above, associations have also been established between gliomas and several rare inheritable syndromes, such as Phakomatoses (neurocutaneous syndromes), Li-Fraumeni, enchondromatosis, and familial polyposis syndromes, which account for <5% of all gliomas (16). Variability in glioma incidence was noted between different ethnic origins, with Caucasian individuals being at higher risk of developing a malignant CNS tumor compared to African-Americans, Asian and Asian/Pacific Islanders (17).

In the United States, Europe, and Australia, glioblastoma is the most commonly reported malignant tumor (14), accounting for 14.2% of all primary brain and other CNS tumors with a particularly low five-year survival rate of ~5 (17). Epidemiologic studies in Canada report similar trends (2, 18, 19), though no detailed analysis by individual cities and specific regions is yet available to demonstrate local trends of CNS malignancies that can help identify putative environmental risk factors.

The primary objective of this study is to describe the geographic distribution of the glioma disease burden in Canada by province, city, and forward sortation area (FSA: first three entries of a postal code). This comprehensive approach to mapping the geographic distribution of CNS cancers may help establish risk factors and identify putative etiologic agents that trigger glioma development.

## Methods

This study was conducted in accordance with the CISS-RDC-668035 and 13-SSH-MCG-3749 protocols approved by the Social Sciences and Humanities Research Council of Canada (SSHRC) and the Quebec Inter-University Centre for Social Statistics (QICSS), respectively as previously described (20–24). In accordance with the institutional policy, this study received McGill University Research Ethics Board exemption.

Clinical and geographic data was obtained from the Canadian Cancer Registry (CCR) and Le Registre Québécois du Cancer (LRQC). The CCR is a dynamic database of cancer patients from 12 provinces and territories (excluding Quebec), who were diagnosed with primary tumors (20–24).

The LRQC database was used to obtain corresponding data for patients residing in Quebec. As data from the LRQC, unfortunately, only spans the years 1992 to 2010, we decided to analyze the data during this time period to encompass all provinces and territories. Geographic and clinical information, including patients’ sex, year of diagnosis, age at the time of diagnosis, and FSA (the first 3 digits of a postal code, which defines a geographical region of residence), as well as the ICD-O-3 code of the tumor, were acquired from the CCR/LRQC databases.

Gliomas comprise ~81% of all malignant brain tumors (14). Consistent with the updated 2016 WHO classification of central nervous system neoplasms, glioma cases were defined based on the International Classification of Diseases for Oncology ICD-O-3 codes according to cancer morphology: Mixed glioma (ICD-O-3 code 9382), Ependymoma, not specified (NOS) (9392), Astrocytoma, NOS (9400), Glioblastoma (9440), Oligodendroglioma, NOS (9450), Oligodendroglioma, anaplastic (9451), Protolasmic astrocytoma (9410), Gemistocytic astrocytoma (9411), Gliomatosis cerebri (9381), Pleomorhic xanthoastrocytoma (9424), Giant cell Glioblastoma (9441), Gliosarcoma (9442), Astroblastoma (9430), Ganglioglioma, NOS (9505), and Pilocytic astrocytoma (9421). For a summary of ICD diagnostic definitions, please refer to **Supplementary Table 1**.

Population counts for incidence were obtained on national, provincial, city and FSA postal code levels from the Canadian Census of Population for the years 1996, 2001, 2006, and 2011 according to Statistics Canada. In Canada, postal codes consist of 6 letters and numbers (*e.g.*, H3G 1A4), where the first 3 entries (*i.e.*, FSA) define a region in the country. There are more than 1,600 FSAs across Canada (23). Annual population counts for Canada from the census data was used to calculate annual incidence of glioma and glioblastoma.

To better elucidate the impact of pollution and industrial risk factors on the incidence of gliomas, significantly higher and lower incidence FSAs were searched using Standard Industrial Classification codes to detect significant industrial activity.

### Mandatory data rounding

In accordance with the CCR, LRQC, and CVS data publication rules, to preserve patient privacy and confidentiality, rounding of variables as absolute numbers for glioma cases was conducted for all values presented in this study. With regards to the rounding of tabular data, SSHRC/Statistics Canada required rounding of each cell count, independent of other cells, to a lower or higher multiple of 5 using an unbiased, random rounding scheme in which counts are more likely to be rounded to their nearest multiple of 5. Counts ≥1 and <5 were restricted from publication, according to the SSHRC regulation. Therefore, if the number of cases were ≥1 but <5 per ICD-O-3 code, then its respective clinical and geographic information could not be released in order to protect patient confidentiality. In order to assess low incidence regional clusters, we were able to review communities with zero recorded glioma cases.

### Statistical and mapping analyses

Unless otherwise specified, analyses of the complete data on all patients with glioma across Canada for the period from 1992 through 2010 are presented throughout this report. Incidence rates and 95% confidence intervals (CIs) were calculated and are reported overall, by the year of diagnosis, and for specific regions identified by the mapping analysis. Unless otherwise specified, Canadian Census averages from the years 1996, 2001, 2006, and 2011 were used for all population analyses. CIs were based on the exact Poisson distribution. Trends over time were assessed using simple regression models and joinpoint regression analyses (25). The coefficient of determination was calculated to establish how closely the incidence rates correspond to the regression line. The joinpoint regression analysis determines the best-fitting regression line and determines whether there are points in time (joinpoints) where significant changes take place.

Geographic maps of Canada divided by city and FSA were generated using ArcGIS Pro mapping software. In mapping the CCR/LRQC results, only regions with populations of at least 10,000 individuals based on 1996, 2001, 2006, and 2010 census data were selected to reduce erroneous false-positive hits, in which a few cases of gliomas occurring within scarcely populated cities and postal codes (<10,000 residents) might have artificially inflated the incidence rate.

## Results

### Demographic characteristics of glioma patients

In Canada, between 1992 and 2010, there were ~32,360 cases of glioma. Of these, 17,115 (52.9%) were glioblastoma. These patients’ clinical characteristics were studied using the demographic information available in two population-based health registries: the CCR and LRQC.

Both total glioma cases and glioblastoma subset cases were analyzed by age and sex to determine the demographic distribution for these malignancies. Our data illustrates that gliomas more commonly affected males (58% males vs. 42% females), which is consistent with the international trends (14). The average age at diagnosis of gliomas was 54.62 years of age (SD = 19.57). However, the highest incidence age group was notably 70-79 years, at 17.55 new glioma cases per 100,000 individuals per year. Studying glioblastoma specifically, male patients were also more affected than female patients (59% males vs. 41% females). The average age of diagnosis was 62.34 years (SD = 13.96). The highest incidence age group remained at 70-79 years, with 12.39 new glioblastoma cases per 100,000 individuals per year.

### Incidence of glioma in Canada during 1992-2010

The overall crude incidence rate of gliomas in Canada during this time period was analyzed, yielding a mean glioma incidence of approximately 5.45 cases per 100,000 individuals per year. Linear regression analysis of the glioma crude incidence rate revealed trend of annual increase of 0.053 ± 0.0050 cases per 100,000 individuals per year.

**Figure 1:**
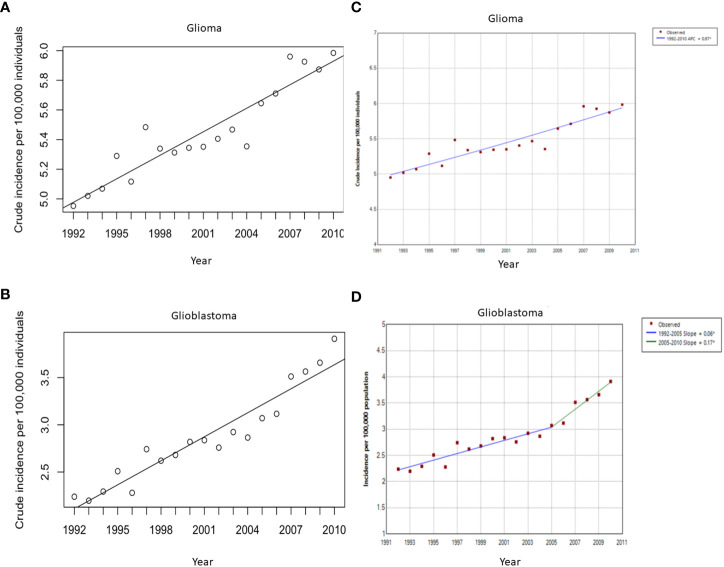
Crude incidence rates (per 100,000 individuals per year) of all glioma **(A)** and glioblastoma multiforme **(B)** cases between 1992 and 2010 with the line of best fit, and linear regression analysis of the incidence rate over time. **(A)** The slope of the line is 0.053 cases per 100,000 individuals per year, p < 0.0001. **(B)** The slope of the line is 0.085 cases per 100,000 individuals per year, p < 0.0001. Joinpoint analysis for incidence for all gliomas **(C)** and Glioblastoma multiforme **(D)**. Change in slope in glioblastoma multiforme incidence was detected after 2005 from 0.06 to 0.17 cases per 100,000 per year.

More specific analysis for glioblastoma incidence in Canada demonstrated a similar consistently increasing trend. The national average for glioblastoma crude incidence was 2.87 cases per 100,000 individuals per year. Linear regression of this incidence from 1992 to 2010 shows an average annual increase of 0.085 ± 0.0064 cases per 100,000 individuals per year (**Figure 1**). Joinpoint analysis of incidence trend was performed for gliomas and glioblastomas. While there were no changes in incidence trends in gliomas, this analysis detected an increase in incidence slope for glioblastomas following 2005 year (**Figure 1**).

### Geographic distribution of glioma cases in Canada

**Figure 2:**
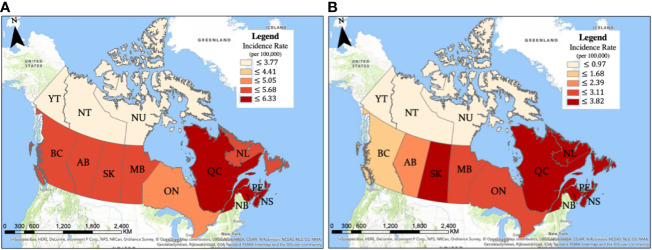
Crude incidence of **(A)** gliomas and **(B)** glioblastomas by province.

The crude glioma incidence rates were analyzed on provincial, city, and FSA postal code levels, revealing distinct areas of higher incidence. As demonstrated by mapping, Quebec (6.33 cases per 100,000/year), Nova scotia (6.21 cases per 100,000/year), and New Brunswick (5.93 cases per 100,000/year) had significantly higher incidences compared to the national average of 5.45 cases per 100,000 individuals per year. On the other hand, provinces and territories with significantly lower glioma incidences included Alberta (5.11 cases per 100,000/year), Ontario (4.95 cases per 100,000/year), Northwest Territories (2.51 cases per 100,000/year), and Nunavut (1.88 cases per 100,000/year) (**Figure 2**; **Table 1**). As mentioned earlier, variability in glioma incidence was noted between different ethnic origins (2, 17). Canadian provinces have different ethnic composition, as detailed in **Supplementary Table 2**, which could in part explain the observed epidemiologic trends.

**Table 1:** Provinces/territories with significantly higher or lower incidence of (A) glioma and (B) glioblastoma.

(A)					
Province	Rounded cases	Reference population	Incidence per 100,000	Upper adjusted CI (95%)	Lower adjusted CI (95%)
Provinces with higher incidence of glioma					
Quebec	8,955	7,446,000	6.330	6.199	6.462
Nova Scotia	1,100	933,000	6.205	5.844	6.583
New Brunswick	845	750,000	5.930	5.537	6.344
Provinces with lower incidence of glioma					
Alberta	3,020	3,109,000	5.112	4.932	5.298
Ontario	11,165	11,868,000	4.951	4.860	5.044
Northwest Territories	20	42,000	2.506	1.530	3.871
Nunavut	10	28,000	1.880	0.900	3.457

(B)					
Province/Territories	Rounded cases	Reference population	Incidence per 100,000	Upper adjusted CI (95%)	Lower adjusted CI (95%)
Provinces with higher incidence of glioblastoma					
Quebec	5,400	7,446,000	3.817	3.716	3.920
New Brunswick	510	750,000	3.579	3.275	3.903
Nova Scotia	610	933,000	3.441	3.173	3.725
Newfound Land and Labrador	340	536,000	3.339	2.993	3.713
Saskatchewan	635	1,010,000	3.309	3.057	3.577
Provinces with lower incidence of glioblastoma					
Alberta	1,400	3,109,000	2.370	2.247	2.498
British Columbia	815	4,040,000	1.062	0.990	1.137
Northwest Territories	5	42,000	0.627	0.202	1.462

Analyses of glioma incidence by city demonstrated a total of 33 cities, where glioma incidences were significantly higher than the national average of 5.45 cases per 100,000 individuals per year. Meanwhile, 73 cities demonstrated significantly lower incidences. **Supplementary Tables 3** and **5** provide detailed incidence of cases.

To better understand the characteristics of regions with higher/lower incidences, we analyzed the incidence of gliomas on a finer level using FSA postal codes across Canada. These analyses revealed 95 FSAs with statistically significant higher incidences. Similarly, glioblastoma incidences by province, city, and FSA were also documented. Full detailed incidences by city and FSA can be found in **Supplementary Tables 4** and **6**.

Standard Industrial Classification codes for significantly higher and lower glioma incidence FSAs were searched to further elucidate correlations with potentially carcinogenic industrial activity. A T-test comparing the means of higher and lower incidence areas, assuming unequal variance, revealed that pests and farms (p = 0.0018), as well as industrial land use (p = 0.0004), were significantly correlated with increased glioma incidences. However, once FSAs were adjusted for land area, only airport operations (p = 0.0010) were found to be significantly related to increased glioma incidence (**Tables 2**, **3**).

**Table 2:** Average number of facilities in higher *vs.* lower incidence FSAs; P-values are the result of a t-test comparing the means assuming unequal variance.

	Higher	Lower	Not Significant	T.TESTS
**Power Plants**	0.25	0.099	0.35	0.14
**Pests and Farms**	0.78	0.29	1.09	0.0018*
**Chimneys**	0.46	0.20	0.85	0.058
**Mining Area**	0.62	0.60	0.71	0.94
**Industrial Land Use**	1.60	0.57	0.99	0.0004*
**Ship Building and Repairing**	1.60	1.25	1.40	0.22
**Airport Operations**	4.06	9.81	3.51	<0.001

*-Denotes statistical significance.

**Table 3:** Average number of facilities, adjusted for area of FSA, in higher *vs.* lower incidence FSAs.

	Higher	Lower	Not Significant	T.TESTS
**Power Plants**	0.0010	0.0001	0.0038	0.24
**Pests and Farms**	0.0073	0.017	0.013	0.36
**Chimneys**	0.0095	0.0064	0.017	0.63
**Mining Area**	0.0019	0.0012	0.0013	0.13
**Industrial Land Use**	0.026	0.015	0.023	0.089
**Ship Building and Repairing**	0.007	0.109	0.010	0.41
**Airport Operations**	0.005	0.001	0.000	0.0010

P-values are the result of a t-test comparing the means assuming unequal variance.

## Discussion

The present study investigated Canadian crude incidence rates of gliomas, with specific subset analysis for glioblastoma. Our findings are consistent with a recent study documenting epidemiology of gliomas/glioblastomas during 2010-2015 years conducted in 4 Canadian Provinces (Manitoba, Ontario, Alberta and British Columbia) (2). Overall, men were found to be more likely than women to have a glioblastoma, as well as to be diagnosed with any glioma. The highest incidence age group for glioblastoma and all gliomas was 70-79, though the average age of diagnosis for both analysis groups was younger. The national average for glioma incidence was 5.45 cases per 100,000/year with an increasing incidence of 0.053 cases per 100,000/year. The incidence of glioblastoma specifically, was 2.87 cases per 100,000/year with an average annual increase of 0.085 cases per 100,000/year. Joinpoint analysis documented a change in incidence slope for glioblastoma after 2005 from 0.06 to 0.17 cases per 100,000 per year. Provincially, Quebec led in glioma/glioblastoma incidence, while the lowest glioma incidence was documented in Nunavut and Northwest Territories. Based on available international data, both glioma and specific glioblastoma subtype findings are consistent with tendencies of incidence increase in the US and Europe (2, 14, 26), suggesting Canada is congruent with international trends in the glioma disease burden.

Currently, the only well-established external risk factors associated with developing gliomas is exposure to ionizing radiation and an inverse relationship to the presence of personal history of atopic dermatitis, rhinitis and other allergies, while exposure to UFPs may be emerging as a novel risk factor for these cancers (12, 13). There is a growing body of literature surrounding “the Cancer Hygiene Hypothesis” (27). In particular, depending on the cancer type, chronic allergic disorders have been connected to both pro and antitumor effects (6, 28, 29). Further research distinguishing the pro and antitumor effects of allergic disorders will help elucidate this relationship. Also, as highlighted earlier, since a number of gliomas may be asymptomatic, increased medical imaging can contribute to higher incidences of gliomas in select urban locations and in specific populations (*i.e.*, individuals who are more likely to seek/receive imaging investigation). Notably, incidence of gliomas is known to vary by ethnicity (with higher incidences being reported in Caucasians) and level of urbanization and socioeconomic status of a given community (26).

According to the Government of Canada’s 2017 Report on Occupational Radiation Exposures (30), approximately 86,000 workers were occupationally exposed to ionizing radiation, with a slight predominance of 53% in male workers. Most exposed workers were nurses, aircrew members, ward aids, and orderlies. However, 80% of all documented ionizing radiation exposures were in low doses (0-1 millisievert or mSv), while the remaining were exposed to moderate doses (1-20mSV), and almost none were exposed to high dose radiation. On a geographic level, the total number of workers with documented ionizing radiation were most concentrated in Ontario (36,000 workers), Quebec (16,000 workers) Alberta (10,000 workers), and British Columbia (9,600 workers). However, once adjusted per-capita of 10,000 workers, the leading provinces were Saskatchewan (111/10,000 workers), New Brunswick (58/10,000 workers), and Ontario (52/10,000 workers). However, the report did not include further analysis on a more regional level and did not permit epidemiological comparison by city or FSA levels. It thus remains possible that pockets of higher ionizing radiation exposure exist within each province. This study heightens the need to investigate environmental and occupational exposures as the cardinal component in the pathogenesis of gliomas.

To better elucidate the impact of pollution and industrial risk factors on the incidence of gliomas, Standard Industrial Classification codes were searched in FSAs with significantly higher and lower glioma incidence. These codes included power plants, pests and farms, chimneys, mining areas, industrial land use, ship building and repairing, and airport operations. This analysis revealed that the presence of pests and farms, as well as industrial land use, was significantly correlated with increased glioma incidences. Different facilities occupy variable areas, and their impact may stretch for miles away from the facility generating pollution, where, for instance, UPSs may impact areas of at least 10 km away from a given major airport (13). Once FSAs were adjusted for land area, only the presence of airport operations was significantly related to increased glioma incidence. Though the risk of increased ionizing radiation exposure for residential areas neighboring airports is yet to be quantified in literature, carcinogenic environmental toxins released by jet fuel combustion, while limited, have previously been established. Though not specifically studied for neurotoxicity, oxidative DNA damage effects were previously demonstrated in peripheral blood lymphocytes and exfoliated buccal cells in airport personnel with long-term jet fuel exposure (31, 32). Geographic findings from this study may also be connected with the outcomes from the Canadian Government’s 2017 Report on Occupational Radiation Exposures findings of predominance of aircrew workers, who may choose to live in the proximity to their workplace. Importantly, as highlighted by a recent study by Wu et al. pollution from UFPs generated at airports may be an important contributor to the increased incidence of gliomas/glioblastomas (13). Other confounding factors must also be accounted for, when discussing living in the proximity to airport operations, such as socioeconomic status and housing standards. Further follow up studies will be necessary to evaluate this important risk factor.

### Limitations

This study had several limitations. Epidemiologic studies are limited by the accuracy of the databases. In particular, this study combined clinical and geographic data from the Canadian Cancer Registry (CCR) and Le Registre Québécois du Cancer (LRQC) in order to determine coherent incidence across time. Although measures have been taken to ensure the quality of data extraction in this study, the risk of data misclassification remains. Importantly, these databases did not provide information on the patients’ ethnicities or family history, both of which are known contributors to developing CNS malignancy. However, studies reveal that monogenic Mendelian disorders comprise only a small percentage of adult glioma incidence on a population level (3, 4), and thus the data analyzed in the present study remains largely reflective of Canadian epidemiology.

Additionally, while mapping by FSA revealed putative clusters, where multiple high-incidence FSAs were located side by side, mapping also revealed instances of low/zero-incidence FSAs located contiguously with reported high-incidence FSAs. This suggests the possible existence of some clusters or sparing by chance. Had these contiguous FSAs been combined, their overall incidence may likely be closer to the national average. Also, considering that glioma and glioblastoma are rare cancers any interpretation of higher/lower incidences in smaller population centers should be made with caution. This is an important limitation of the study. Also, as a number of gliomas may be asymptomatic, increased medical imaging in urban centers leading to incidental diagnosis of early-stage disease, might be affecting the reported numbers. Future investigations for possible glioma incidences should thus focus on true cluster areas of combined high incidence. Finally, this study reports crude incidence and not age-adjusted incidence, which was not possible to calculate due to low number of cases in select areas.

## Conclusion

In conclusion, this epidemiologic study reveals a national glioma incidence rate of 5.45 cases per 100,000 individuals per year with demonstrated crude incidence increase over time. A male predominance was observed in incidence of gliomas/glioblastomas. Provincially, Quebec led in incidence, while the lowest glioma incidence rates were documented in Nunavut and Northwest Territories. A putative regional clustering of gliomas was observed, with higher incidence rates in FSAs with industrial activity related to airport operations.

## Data availability statement

The original contributions presented in the study are included in the article/**Supplementary Material**. Further inquiries can be directed to the corresponding authors.

## Ethics statement

The presented study was conducted in accordance with protocols approved by the Social Sciences and Humanities Research Council of Canada (SSHRC) and the Québec Inter-University Centre for Social Statistics (QICSS), respectively, protocol numbers: CISS-RDC-668035 and 13-SSH-MCG-3749-S001. Further, in accordance with the institutional policy, this study received an exemption from the McGill University Research Ethics Board review.

## Author contributions

Conceptualization, IL and OL. Methodology, XJ, AA, FG, MT, AZ, OL, and IL. Software, XJ and AA. Validation, XJ, AA, FG, AZ, and IL. Formal analysis, XJ, AA, and FG. Investigation, XJ, AA, FG, MT, AZ, OL, and IL. Data curation, XJ. Writing—original draft preparation, XJ. Writing—review and editing, XJ, AA, FG, MT, AZ, OL, and IL. Visualization, XJ and AA. Supervision, OL and IL. Project administration, IL. All authors contributed to the article and approved the submitted version.
