# Supplementary material for: Investigation of incidence and geographic distribution of gliomas in Canada from 1992 to 2010: a national population-based study highlighting the importance of exposure to airport operations
Source: Front Oncol. 2023 May 16;13:1190366. doi: 10.3389/fonc.2023.1190366 (PMC10228722; doi:10.3389/fonc.2023.1190366)
Supplement: Supplementary file 1 [file Table_1.docx]

**Supplementary Materials**

**Supplementary Table 1**: List of the tumors of the central nervous system, including names of the objects of analysis (cancer types) and their corresponding International Classification of Diseases for Oncology, 3^rd^ edition (ICD-O-3) codes.

| **ICD-O-3** | **Name of cancer type** |
| --- | --- |
| **Astrocytic and oligodendroglial tumours** | |
| 9440/3 | Glioblastoma |
| 9041/3 | Astrocytoma, anaplastic |
| 9424/3 | Pheomorphic xanthoastrocytoma |
| 9430/3 | Astroblastoma |
| 9400/3 | Astrocystoma, NOS |
| 9431/1 | Angiocentric glioma |
| 9450/3 | Oligodendroglioma, NOS |
| 9382/3 | Mixed glioma |
| **Ependymal and choroid plexus tumours** | |
| 9391/3 | Ependymoma, NOS |
| 9490/3 | Choroid plexus carcinoma |
| **Tumours of the pineal region** | |
| 9362/3 | Pineoblastoma |
| **Embryonal tumours** | |
| 9470/3 | Medulloblastoma, NOS |
| 9508/3 | Atypical teratoid/rhabdoid tumour |
| **Primary central nervous system lymphomas (PCNSL)** | |
| 9930/3 | Myeloid sarcoma |
| **Germ cell tumours** | |
| 9064/3 | Germinoma |
| 9070/3 | Embryonal carcinoma, NOS |
| 9071/3 | Yolk sac tumour |
| 9100/3 | Chloriocarcinoma, NOS |
| 9080/3 | Teratoma, malignant, NOS |
| 9084/3 | Teratoma with malignant transformation |
| 9085/3 | Mixed germ cell tumour |
| **Tumours of cranial nerves** | |
| 9540/3 | Malignant peripheral nerve sheath tumour |
| **Meningiomas** | |
| 9530/3 | Meningioma, malignant |
| **Mesenchymal non-meningothelial tumours** | |
| 9150/1 | Hemangiopericytoma, NOS |
| 9133/1 | Epithelioid hemangioendothelioma, NOS |
| 9120/3 | Hemangiosarcoma |
| 9140/3 | Kaposi sarcoma |
| 8850/3 | Liposarcoma, NOS |
| 8810/3 | Fibrosarcoma, NOS |
| 8830/3 | Malignant fibrous histiocytoma |
| 8900/3 | Rhabdomyosarcoma, NOS |
| 9220/3 | Chondrosarcoma, NOS |
| 9180/3 | Osteosarcoma, NOS |
| **Melanocytic lesions** | |
| 8720/3 | Malignant melanoma, NOS |
| 8728/3 | Meningeal melanomatosis |

**Supplementary Table 2:** Analysis of the percentage of multiple ethnic origin of Canadians in all provinces and territories, as self-declared by respondents to the 2006 Canadian Census. The British Isles include persons of Cornish, English, Irish, Manx, Scottish and Welsh descent. Other European origins are listed amongst ‘European’ (excludes British Isles and includes French origins). Asian origins include Oceania origins. Note that a respondent in the census survey may report more than one ethnic origin, which explains the sum of rows exceed 100 percent. Data was obtained and analyzed from the archived 2006 Canadian Census Data Program: https://www12.statcan.gc.ca/census-recensement/2006/dp-pd/prof/rel/index-eng.cfm

| Ethnic origin | **Canada** | **British Colombia** | **Alberta** | **Saskatchewan** | **Manitoba** | **Ontario** | **Quebec** | **Newfoundland and Labrador** | **Nova Scotia** | **Prince Edward Island** | **New Brunswick** | **Yukon Territory** | **Northwest Territories** | **Nunavut Territories** |
| --- | --- | --- | --- | --- | --- | --- | --- | --- | --- | --- | --- | --- | --- | --- |
| **British Isles** | 35.5 | 45.7 | 45.7 | 43.1 | 38.0 | 40.9 | 9.6 | 57.0 | 60.0 | 68.1 | 46.1 | 51.1 | 32.1 | 14.4 |
| **European** | 47.8 | 48.0 | 60.7 | 69.2 | 63.3 | 47.7 | 41.7 | 10.0 | 39.5 | 35.5 | 39.6 | 54.5 | 32.4 | 8.5 |
| **Aboriginal** | 5.4 | 6.2 | 7.5 | 15.7 | 16.4 | 3.3 | 3.6 | 7.4 | 5.3 | 2.7 | 4.9 | 25.9 | 69.3 | 85.9 |
| **Caribbean or Latin America** | 3.0 | 1.4 | 1.4 | 0.0 | 1.7 | 4.6 | 3.2 | 0.2 | 1.7 | 0.2 | 0.3 | 0.7 | 0.6 | 0.2 |
| **African** | 1.3 | 0.8 | 1.3 | 0.5 | 1.5 | 2.0 | 1.0 | 0.2 | 1.2 | 0.3 | 0.4 | 0.4 | 0.9 | 0.5 |
| **Arab** | 1.5 | 0.5 | 1.2 | 0.3 | 0.4 | 1.5 | 2.7 | 0.3 | 1.2 | 0.7 | 5.3 | 0.2 | 0.4 | 0.0 |
| **Asian** | 12.4 | 7.3 | 11.5 | 2.6 | 7.4 | 15.8 | 4.3 | 0.9 | 1.7 | 0.8 | 1.2 | 4.3 | 4.6 | 0.9 |
| **Canadian or American** | 33.3 | 18.8 | 21.8 | 19.0 | 18.7 | 23.7 | 62.1 | 49.1 | 41.4 | 39.3 | 53.4 | 21.6 | 15.9 | 4.4 |

**Supplementary Table 3:** (a) Cities with significantly higher incidence of glioma; (b) Cities with significantly lower incidence of glioma. Populations centers with >50,000 residents are highlighted in **bold**.

| City | Reference population | Incidence per 100,000 | Upper adjusted CI (95%) | Lower adjusted CI (95%) |
| --- | --- | --- | --- | --- |
| Alma | 30,000 | 7.895 | 5.758 | 10.564 |
| Annapolis | 26,000 | 8.097 | 5.784 | 11.026 |
| Bathurst | 13,000 | 10.121 | 6.548 | 14.942 |
| Boucherville | 40,000 | 7.895 | 6.024 | 10.162 |
| Central Saanich | 15,000 | 8.772 | 5.675 | 12.950 |
| Chateauguay | 43,000 | 7.956 | 6.140 | 10.141 |
| Dorval | 18,000 | 8.772 | 5.917 | 12.523 |
| Gaspe | 15,000 | 8.772 | 5.675 | 12.950 |
| Joliette | 19,000 | 8.310 | 5.606 | 11.864 |
| Kings | 47,000 | 8.399 | 6.606 | 10.528 |
| **Langley** | **91,000** | **6.940** | **5.754** | **8.299** |
| **Laval** | **361,000** | **6.342** | **5.760** | **6.967** |
| Mercier | 10,000 | 10.526 | 6.427 | 16.258 |
| **Moncton** | **63,000** | **7.937** | **6.421** | **9.702** |
| Moose Jaw | 33,000 | 7.974 | 5.918 | 10.514 |
| North Saanich | 11,000 | 9.569 | 5.843 | 14.780 |
| Oak Bay | 18,000 | 13.158 | 9.597 | 17.607 |
| Parksville | 11,000 | 9.569 | 5.843 | 14.780 |
| Pictou | 21,000 | 8.772 | 6.109 | 12.200 |
| Portage La Prairie | 13,000 | 12.146 | 8.193 | 17.340 |
| Rimouski | 46,000 | 7.437 | 5.739 | 9.479 |
| **Saanich** | **106,000** | **6.703** | **5.620** | **7.934** |
| Saint-Antoine | 11,000 | 9.569 | 5.843 | 14.780 |
| Saint-Hyacinthe | 39,000 | 8.097 | 6.179 | 10.423 |
| Saint-Lambert | 22,000 | 8.373 | 5.831 | 11.646 |
| Sainte-Therese | 25,000 | 8.421 | 6.015 | 11.467 |
| Sidney | 11,000 | 9.569 | 5.843 | 14.780 |
| Squamish | 15,000 | 8.772 | 5.675 | 12.950 |
| Summerland | 11,000 | 9.569 | 5.843 | 14.780 |
| **Thunder Bay** | **11,6000** | **6.806** | **5.760** | **7.986** |
| Tillsonburg | 14,000 | 9.398 | 6.081 | 13.875 |
| **Victoria** | **76,000** | **6.925** | **5.634** | **8.423** |
| Westmount | 20,000 | 9.211 | 6.414 | 12.810 |
| White Rock | 18,000 | 8.772 | 5.917 | 12.523 |
| Yarmouth | 10,000 | 15.789 | 10.651 | 22.541 |

| City | Reference population | Incidence per 100,000 | Upper adjusted CI (95%) | Lower adjusted CI (95%) |
| --- | --- | --- | --- | --- |
| **Ajax** | **84,000** | **3.759** | **2.869** | **4.839** |
| Anjou | 38,000 | 3.463 | 2.240 | 5.112 |
| Aurora | 44,000 | 3.589 | 2.421 | 5.123 |
| Bellefeuille | 13,000 | 2.024 | 0.652 | 4.724 |
| Belleville | 48,000 | 3.838 | 2.673 | 5.338 |
| **Brampton** | **388,000** | **3.459** | **3.047** | **3.911** |
| **Burnaby** | **200,000** | **4.342** | **3.705** | **5.058** |
| **Calgary** | **933,000** | **5.021** | **4.696** | **5.362** |
| **Cambridge** | **115,000** | **3.432** | **2.700** | **4.303** |
| Cap-Rouge | 14,000 | 1.880 | 0.606 | 4.387 |
| Central Okanagan H | 15,000 | 1.754 | 0.565 | 4.094 |
| Centre Wellington | 25,000 | 2.105 | 1.008 | 3.872 |
| **Chatham-Kent** | **107,000** | **3.197** | **2.467** | **4.075** |
| **Coquitlam** | **114,000** | **4.386** | **3.548** | **5.362** |
| **Dartmouth** | **68,000** | **1.935** | **1.252** | **2.857** |
| **Drummondville** | **70,000** | **3.383** | **2.468** | **4.527** |
| **Edmonton** | **706,000** | **4.697** | **4.337** | **5.078** |
| Ennismore | 17,000 | 1.548 | 0.499 | 3.612 |
| Essex | 20,000 | 2.632 | 1.260 | 4.840 |
| **Etobicoke** | **365,000** | **2.451** | **2.097** | **2.849** |
| **Gatineau** | **572,000** | **1.748** | **1.508** | **2.015** |
| **Halifax** | **366,000** | **4.314** | **3.840** | **4.831** |
| **Hamilton** | **496,000** | **4.563** | **4.142** | **5.015** |
| **Kawartha Lakes** | **71,000** | **4.077** | **3.071** | **5.307** |
| Kingsville | 20,000 | 2.632 | 1.260 | 4.840 |
| Lakeshore | 31,000 | 2.547 | 1.424 | 4.201 |
| Les Îles-De-La-Madeleine | 12,000 | 2.193 | 0.707 | 5.118 |
| **Lévis** | **134,000** | **1.964** | **1.457** | **2.589** |
| **Longueuil** | **230,000** | **4.005** | **3.433** | **4.644** |
| Loyalist | 15,000 | 1.754 | 0.565 | 4.094 |
| **Markham** | **236,000** | **3.568** | **3.037** | **4.166** |
| **Milton** | **50,000** | **3.684** | **2.566** | **5.124** |
| Mirabel | 32,000 | 3.289 | 2.008 | 5.081 |
| **Mississauga** | **635,000** | **4.061** | **3.710** | **4.437** |
| **Montreal** | **1,723,000** | **4.338** | **4.115** | **4.569** |
| **Norfolk** | **62,000** | **2.971** | **2.069** | **4.132** |
| **Oshawa** | **141,000** | **4.106** | **3.375** | **4.949** |
| **Ottawa** | **798,000** | **4.287** | **3.964** | **4.630** |
| Perth | 30,000 | 2.632 | 1.472 | 4.341 |
| **Pierrefonds** | **54,000** | **3.899** | **2.785** | **5.309** |
| Port Alberni | 18,000 | 2.924 | 1.400 | 5.378 |
| Port Hope And Hope | 16,000 | 1.645 | 0.530 | 3.838 |
| **Prince George** | **73,000** | **3.965** | **2.987** | **5.162** |
| **Québec** | **504,000** | **4.125** | **3.728** | **4.552** |
| Quinte West | 42,000 | 2.506 | 1.530 | 3.871 |
| **Richmond** | **170,000** | **3.870** | **3.221** | **4.611** |
| **Richmond Hill** | **146,000** | **3.785** | **3.096** | **4.582** |
| Rock Forest | 18,000 | 1.462 | 0.471 | 3.412 |
| **Saguenay** | **144,000** | **2.376** | **1.833** | **3.028** |
| **Saint-Hubert** | **76,000** | **4.155** | **3.171** | **5.349** |
| **Saint-Jean-Sur-Richelieu** | **90,000** | **4.094** | **3.191** | **5.172** |
| **Saint-Jerome** | **90,000** | **2.632** | **1.919** | **3.521** |
| Saint-Lin - Laurentides | 14,000 | 1.880 | 0.606 | 4.387 |
| **Scarborough** | **559,000** | **1.789** | **1.544** | **2.062** |
| **Shawinigan** | **63,000** | **3.759** | **2.742** | **5.031** |
| **Sherbrooke** | **151,000** | **4.531** | **3.786** | **5.380** |
| South Glengarry | 13,000 | 2.024 | 0.652 | 4.724 |
| **Stoney Creek** | **69,000** | **1.907** | **1.234** | **2.815** |
| **Sudbury** | **160,000** | **4.276** | **3.573** | **5.078** |
| Summerside | 15,000 | 1.754 | 0.565 | 4.094 |
| **Surrey** | **379,000** | **4.374** | **3.905** | **4.885** |
| **Terrebonne** | **101,000** | **3.387** | **2.614** | **4.317** |
| Thorold | 18,000 | 2.924 | 1.400 | 5.378 |
| **Toronto** | **2,496,000** | **3.121** | **2.964** | **3.284** |
| **Trois-Rivières** | **152,000** | **4.501** | **3.761** | **5.345** |
| Val-Belair | 21,000 | 2.506 | 1.200 | 4.609 |
| Vaudreuil-Dorion | 24,000 | 2.193 | 1.050 | 4.033 |
| **Vaughan** | **210,000** | **4.135** | **3.528** | **4.817** |
| Wasaga Beach | 13,000 | 2.024 | 0.652 | 4.724 |
| West Grey | 12,000 | 2.193 | 0.707 | 5.118 |
| Wood Buffalo | 48,000 | 3.289 | 2.219 | 4.696 |
| Yellowknife | 18,000 | 2.924 | 1.400 | 5.378 |
| **York** | **845,000** | **2.429** | **2.194** | **2.683** |

**Supplementary Table 4:** FSAs with significantly higher incidence of glioma

| FSA | Rounded Raw Incidence | Reference Population | Incidence per 100,000 | Adjusted upper CI (95%) | Adjusted lower CI (95%) |
| --- | --- | --- | --- | --- | --- |
| G8Z | 30 | 13,000 | 12.146 | 8.193 | 17.340 |
| H3Z | 25 | 12,000 | 10.965 | 7.094 | 16.187 |
| V8S | 35 | 17,000 | 10.836 | 7.546 | 15.071 |
| B4N | 35 | 17,000 | 10.836 | 7.546 | 15.071 |
| G6G | 40 | 20,000 | 10.526 | 7.519 | 14.334 |
| G1W | 40 | 20,000 | 10.526 | 7.519 | 14.334 |
| B2H | 30 | 15,000 | 10.526 | 7.101 | 15.028 |
| K2A | 30 | 15,000 | 10.526 | 7.101 | 15.028 |
| J8C | 20 | 10,000 | 10.526 | 6.427 | 16.258 |
| E1E | 20 | 10,000 | 10.526 | 6.427 | 16.258 |
| T4J | 20 | 10,000 | 10.526 | 6.427 | 16.258 |
| G5L | 60 | 31,000 | 10.187 | 7.773 | 13.113 |
| J4P | 20 | 11,000 | 9.569 | 5.843 | 14.780 |
| G2A | 25 | 14,000 | 9.398 | 6.081 | 13.875 |
| B4H | 25 | 14,000 | 9.398 | 6.081 | 13.875 |
| J0E | 85 | 48,000 | 9.320 | 7.444 | 11.525 |
| G6P | 60 | 34,000 | 9.288 | 7.087 | 11.956 |
| G0T | 30 | 17,000 | 9.288 | 6.265 | 13.260 |
| V8P | 30 | 17,000 | 9.288 | 6.265 | 13.260 |
| G0V | 30 | 17,000 | 9.288 | 6.265 | 13.260 |
| R3P | 30 | 17,000 | 9.288 | 6.265 | 13.260 |
| J6K | 35 | 20,000 | 9.211 | 6.414 | 12.810 |
| H7G | 35 | 20,000 | 9.211 | 6.414 | 12.810 |
| H4G | 50 | 29,000 | 9.074 | 6.735 | 11.964 |
| G1H | 50 | 29,000 | 9.074 | 6.735 | 11.964 |
| G0M | 60 | 36,000 | 8.772 | 6.693 | 11.291 |
| P7B | 50 | 30,000 | 8.772 | 6.510 | 11.565 |
| G7X | 45 | 27,000 | 8.772 | 6.398 | 11.738 |
| S0H | 40 | 24,000 | 8.772 | 6.266 | 11.945 |
| G8B | 40 | 24,000 | 8.772 | 6.266 | 11.945 |
| G5Y | 40 | 24,000 | 8.772 | 6.266 | 11.945 |
| V8N | 40 | 24,000 | 8.772 | 6.266 | 11.945 |
| J0C | 35 | 21,000 | 8.772 | 6.109 | 12.200 |
| H1B | 35 | 21,000 | 8.772 | 6.109 | 12.200 |
| R0M | 35 | 21,000 | 8.772 | 6.109 | 12.200 |
| V9P | 35 | 21,000 | 8.772 | 6.109 | 12.200 |
| B0S | 30 | 18,000 | 8.772 | 5.917 | 12.523 |
| G1K | 30 | 18,000 | 8.772 | 5.917 | 12.523 |
| V1E | 30 | 18,000 | 8.772 | 5.917 | 12.523 |
| G1M | 30 | 18,000 | 8.772 | 5.917 | 12.523 |
| J1J | 25 | 15,000 | 8.772 | 5.675 | 12.950 |
| A0C | 25 | 15,000 | 8.772 | 5.675 | 12.950 |
| J4H | 25 | 15,000 | 8.772 | 5.675 | 12.950 |
| V7V | 25 | 15,000 | 8.772 | 5.675 | 12.950 |
| C1A | 50 | 31,000 | 8.489 | 6.300 | 11.192 |
| J3G | 45 | 28,000 | 8.459 | 6.169 | 11.319 |
| J8Y | 40 | 25,000 | 8.421 | 6.015 | 11.467 |
| V8L | 35 | 22,000 | 8.373 | 5.831 | 11.646 |
| H8P | 35 | 22,000 | 8.373 | 5.831 | 11.646 |
| H9S | 35 | 22,000 | 8.373 | 5.831 | 11.646 |
| B0K | 60 | 38,000 | 8.310 | 6.341 | 10.697 |
| J4B | 60 | 38,000 | 8.310 | 6.341 | 10.697 |
| J6E | 60 | 38,000 | 8.310 | 6.341 | 10.697 |
| V4B | 30 | 19,000 | 8.310 | 5.606 | 11.864 |
| H4H | 30 | 19,000 | 8.310 | 5.606 | 11.864 |
| J0G | 30 | 19,000 | 8.310 | 5.606 | 11.864 |
| V0H | 80 | 51,000 | 8.256 | 6.546 | 10.275 |
| T2V | 50 | 32,000 | 8.224 | 6.103 | 10.842 |
| V1Y | 50 | 32,000 | 8.224 | 6.103 | 10.842 |
| B2N | 45 | 29,000 | 8.167 | 5.956 | 10.928 |
| P7C | 45 | 29,000 | 8.167 | 5.956 | 10.928 |
| J7E | 45 | 29,000 | 8.167 | 5.956 | 10.928 |
| H4A | 40 | 26,000 | 8.097 | 5.784 | 11.026 |
| K1J | 40 | 26,000 | 8.097 | 5.784 | 11.026 |
| G1G | 35 | 23,000 | 8.009 | 5.578 | 11.139 |
| H3X | 35 | 23,000 | 8.009 | 5.578 | 11.139 |
| G1L | 35 | 23,000 | 8.009 | 5.578 | 11.139 |
| J2W | 35 | 23,000 | 8.009 | 5.578 | 11.139 |
| V3A | 60 | 40,000 | 7.895 | 6.024 | 10.162 |
| S6H | 45 | 30,000 | 7.895 | 5.758 | 10.564 |
| H4E | 45 | 30,000 | 7.895 | 5.758 | 10.564 |
| H1T | 45 | 30,000 | 7.895 | 5.758 | 10.564 |
| G0R | 125 | 84,000 | 7.832 | 6.519 | 9.332 |
| V0R | 95 | 64,000 | 7.813 | 6.321 | 9.551 |
| L0N | 40 | 27,000 | 7.797 | 5.570 | 10.618 |
| B3M | 40 | 27,000 | 7.797 | 5.570 | 10.618 |
| B0P | 65 | 44,000 | 7.775 | 6.000 | 9.910 |
| G1C | 50 | 34,000 | 7.740 | 5.744 | 10.204 |
| J6A | 50 | 34,000 | 7.740 | 5.744 | 10.204 |
| B0J | 70 | 48,000 | 7.675 | 5.983 | 9.698 |
| V0N | 110 | 77,000 | 7.519 | 6.179 | 9.062 |
| G0X | 90 | 63,000 | 7.519 | 6.046 | 9.242 |
| J0R | 70 | 49,000 | 7.519 | 5.861 | 9.500 |
| V2A | 50 | 35,000 | 7.519 | 5.580 | 9.913 |
| G6V | 50 | 35,000 | 7.519 | 5.580 | 9.913 |
| S7H | 50 | 35,000 | 7.519 | 5.580 | 9.913 |
| G0A | 135 | 95,000 | 7.479 | 6.271 | 8.853 |
| L0G | 55 | 39,000 | 7.422 | 5.591 | 9.662 |
| J0H | 75 | 54,000 | 7.310 | 5.749 | 9.163 |
| T0J | 65 | 47,000 | 7.279 | 5.617 | 9.278 |
| G0C | 70 | 52,000 | 7.085 | 5.523 | 8.952 |
| T0M | 85 | 64,000 | 6.990 | 5.583 | 8.644 |
| J0L | 100 | 77,000 | 6.835 | 5.561 | 8.314 |
| J0K | 170 | 132,000 | 6.778 | 5.798 | 7.877 |
| K0K | 130 | 103,000 | 6.643 | 5.550 | 7.888 |

**Supplementary Table 5:** (a) Cities with significantly higher incidence of glioblastoma; (b) Cities with significantly lower incidence of glioblastoma. Populations centers with >50,000 residents are highlighted in **bold**.

| City | Rounded Raw Incidence | Reference Population | Incidence per 100,000 | Adjusted lower IC (95%) | Adjusted upper IC (95%) |
| --- | --- | --- | --- | --- | --- |
| Bathurst | 15 | 13,000 | 6.073 | 3.396 | 10.017 |
| Beloeil | 20 | 20,000 | 5.263 | 3.214 | 8.129 |
| Boucherville | 35 | 40,000 | 4.605 | 3.207 | 6.405 |
| Chateauguay | 40 | 43,000 | 4.896 | 3.497 | 6.667 |
| Conception Bay South | 20 | 21,000 | 5.013 | 3.060 | 7.742 |
| Dorval | 25 | 18,000 | 7.310 | 4.729 | 10.791 |
| Gaspe | 20 | 15,000 | 7.018 | 4.285 | 10.839 |
| Joliette | 20 | 19,000 | 5.540 | 3.383 | 8.557 |
| King | 20 | 19,000 | 5.540 | 3.383 | 8.557 |
| Kings | 40 | 42,000 | 5.013 | 3.581 | 6.826 |
| La Tuque | 15 | 12,000 | 6.579 | 3.679 | 10.852 |
| Lambton Shores | 15 | 11,000 | 7.177 | 4.014 | 11.838 |
| **London** | **235** | **345,000** | **3.585** | **3.141** | **4.074** |
| Mercier | 15 | 10,000 | 7.895 | 4.415 | 13.022 |
| **Moncton** | **55** | **63,000** | **4.595** | **3.461** | **5.981** |
| Moose Jaw | 30 | 33,000 | 4.785 | 3.228 | 6.831 |
| Owen Sound | 25 | 22,000 | 5.981 | 3.869 | 8.829 |
| Pictou | 25 | 21,000 | 6.266 | 4.054 | 9.250 |
| Rimouski | 45 | 46,000 | 5.149 | 3.755 | 6.890 |
| Saint-Hyacinthe | 35 | 39,000 | 4.723 | 3.289 | 6.569 |
| Saint-Lambert | 20 | 21,000 | 5.013 | 3.060 | 7.742 |
| Sandwich West | 25 | 26,000 | 5.061 | 3.274 | 7.471 |
| **Saskatoon** | **140** | **204,000** | **3.612** | **3.038** | **4.262** |
| Thetford Mines | 25 | 26,000 | 5.061 | 3.274 | 7.471 |
| **Thunder Bay** | **90** | **110,000** | **4.306** | **3.463** | **5.293** |
| Tillsonburg | 15 | 14,000 | 5.639 | 3.154 | 9.301 |
| Westmount | 25 | 20,000 | 6.579 | 4.256 | 9.712 |
| **York** | **255** | **255,000** | **5.263** | **4.637** | **5.950** |

| City | Rounded Raw Incidence | Reference Population | Incidence per 100,000 | Adjusted lower IC (95%) | Adjusted upper IC (95%) |
| --- | --- | --- | --- | --- | --- |
| **Abbotsford** | **20** | **120,000** | **0.877** | **0.536** | **1.355** |
| Anjou | 10 | 38,000 | 1.385 | 0.663 | 2.547 |
| **Brampton** | **135** | **388,000** | **1.831** | **1.535** | **2.168** |
| **Burnaby** | **30** | **200,000** | **0.789** | **0.533** | **1.127** |
| **Cambridge** | **45** | **115,000** | **2.059** | **1.502** | **2.756** |
| Centre Wellington | 5 | 25,000 | 1.053 | 0.339 | 2.456 |
| **Chilliwack** | **10** | **68,000** | **0.774** | **0.371** | **1.424** |
| **Coquitlam** | **20** | **114,000** | **0.923** | **0.564** | **1.426** |
| **Dartmouth** | **10** | **68,000** | **0.774** | **0.371** | **1.424** |
| **Delta** | **20** | **97,000** | **1.085** | **0.663** | **1.676** |
| **Edmonton** | **225** | **706,000** | **1.677** | **1.465** | **1.911** |
| **Etobicoke** | **100** | **365,000** | **1.442** | **1.173** | **1.754** |
| **Gatineau** | **110** | **318,000** | **1.821** | **1.496** | **2.194** |
| **Kamloops** | **15** | **80,000** | **0.987** | **0.552** | **1.628** |
| **Kelowna** | **35** | **103,000** | **1.788** | **1.246** | **2.487** |
| **Langley** | **20** | **91,000** | **1.157** | **0.706** | **1.787** |
| **Lévis** | **30** | **134,000** | **1.178** | **0.795** | **1.682** |
| **Longueuil** | **100** | **230,000** | **2.288** | **1.862** | **2.783** |
| **Maple Ridge** | **15** | **66,000** | **1.196** | **0.669** | **1.973** |
| **Markham** | **95** | **236,000** | **2.119** | **1.714** | **2.590** |
| **Mississauga** | **265** | **635,000** | **2.196** | **1.940** | **2.477** |
| **Montréal** | **845** | **1,718,000** | **2.589** | **2.417** | **2.769** |
| **Nanaimo** | **20** | **76,000** | **1.385** | **0.846** | **2.139** |
| **New Westminster** | **10** | **57,000** | **0.923** | **0.442** | **1.698** |
| **Port Coquitlam** | **5** | **52,000** | **0.506** | **0.163** | **1.181** |
| **Prince George** | **10** | **73,000** | **0.721** | **0.345** | **1.326** |
| **Richmond** | **15** | **170,000** | **0.464** | **0.260** | **0.766** |
| **Richmond Hill** | **55** | **146,000** | **1.983** | **1.494** | **2.581** |
| **Saanich** | **25** | **106,000** | **1.241** | **0.803** | **1.833** |
| **Saguenay** | **30** | **144,000** | **1.096** | **0.740** | **1.565** |
| Saint-Georges | 5 | 30,000 | 0.877 | 0.283 | 2.047 |
| **Saint-Hubert** | **25** | **76,000** | **1.731** | **1.120** | **2.556** |
| **Saint-Jérôme** | **15** | **66,000** | **1.196** | **0.669** | **1.973** |
| **Scarborough** | **105** | **559,000** | **0.989** | **0.809** | **1.197** |
| Sept-Îles | 5 | 26,000 | 1.012 | 0.326 | 2.362 |
| **Stoney Creek** | **10** | **54,000** | **0.975** | **0.467** | **1.793** |
| **Strathcona County No. 20** | **15** | **78,000** | **1.012** | **0.566** | **1.669** |
| **Surrey** | **50** | **379,000** | **0.694** | **0.515** | **0.915** |
| **Terrebonne** | **40** | **101,000** | **2.084** | **1.489** | **2.838** |
| **Toronto** | **935** | **2,496,000** | **1.972** | **1.847** | **2.102** |
| **Vancouver** | **100** | **685,000** | **0.768** | **0.625** | **0.935** |
| Vernon | 10 | 35,000 | 1.504 | 0.720 | 2.766 |
| **Victoria** | **25** | **76,000** | **1.731** | **1.120** | **2.556** |
| Wood Buffalo | 10 | 48,000 | 1.096 | 0.525 | 2.017 |

**Supplementary Table 6:** (a) FSAs with significantly higher incidence of glioblastoma; (b) FSAs with significantly lower incidence of glioblastoma.

| FSA | Rounded Raw Incidence | Reference Population | Incidence per 100,000 | Adjusted upper CI (95%) | Adjusted lower CI (95%) |
| --- | --- | --- | --- | --- | --- |
| G1W | 30 | 20,000 | 7.89 | 5.33 | 11.27 |
| G6G | 30 | 20,000 | 7.89 | 5.33 | 11.27 |
| J8C | 15 | 10,000 | 7.89 | 4.42 | 13.02 |
| E1E | 15 | 10,000 | 7.89 | 4.42 | 13.02 |
| G1K | 25 | 18,000 | 7.31 | 4.73 | 10.79 |
| N6A | 15 | 11,000 | 7.18 | 4.02 | 11.84 |
| J4P | 15 | 11,000 | 7.18 | 4.02 | 11.84 |
| J1J | 20 | 15,000 | 7.02 | 4.29 | 10.84 |
| G5L | 40 | 31,000 | 6.79 | 4.85 | 9.25 |
| K1H | 20 | 16,000 | 6.58 | 4.02 | 10.16 |
| G9X | 15 | 12,000 | 6.58 | 3.68 | 10.85 |
| H3Z | 15 | 12,000 | 6.58 | 3.68 | 10.85 |
| N7V | 15 | 12,000 | 6.58 | 3.68 | 10.85 |
| G1H | 35 | 29,000 | 6.35 | 4.42 | 8.83 |
| B4N | 20 | 17,000 | 6.19 | 3.78 | 9.56 |
| G1R | 20 | 17,000 | 6.19 | 3.78 | 9.56 |
| K1L | 20 | 17,000 | 6.19 | 3.78 | 9.56 |
| G8Z | 15 | 13,000 | 6.07 | 3.40 | 10.02 |
| L6K | 15 | 13,000 | 6.07 | 3.40 | 10.02 |
| N2C | 15 | 13,000 | 6.07 | 3.40 | 10.02 |
| H9S | 25 | 22,000 | 5.98 | 3.87 | 8.83 |
| G0M | 40 | 36,000 | 5.85 | 4.18 | 7.96 |
| G1M | 20 | 18,000 | 5.85 | 3.57 | 9.03 |
| T2V | 35 | 32,000 | 5.76 | 4.01 | 8.01 |
| H3X | 25 | 23,000 | 5.72 | 3.70 | 8.45 |
| G2A | 15 | 14,000 | 5.64 | 3.16 | 9.30 |
| G9N | 20 | 19,000 | 5.54 | 3.38 | 8.56 |
| J0A | 20 | 19,000 | 5.54 | 3.38 | 8.56 |
| J0G | 20 | 19,000 | 5.54 | 3.38 | 8.56 |
| M8Y | 20 | 19,000 | 5.54 | 3.38 | 8.56 |
| J0E | 50 | 48,000 | 5.48 | 4.07 | 7.23 |
| N5W | 25 | 24,000 | 5.48 | 3.55 | 8.09 |
| G1X | 25 | 24,000 | 5.48 | 3.55 | 8.09 |
| S0H | 25 | 24,000 | 5.48 | 3.55 | 8.09 |
| H1R | 30 | 29,000 | 5.44 | 3.67 | 7.77 |
| H4G | 30 | 29,000 | 5.44 | 3.67 | 7.77 |
| G6P | 35 | 34,000 | 5.42 | 3.77 | 7.54 |
| L0G | 40 | 39,000 | 5.40 | 3.86 | 7.35 |
| N0H | 45 | 45,000 | 5.26 | 3.84 | 7.04 |
| G6V | 35 | 35,000 | 5.26 | 3.67 | 7.32 |
| S7H | 35 | 35,000 | 5.26 | 3.67 | 7.32 |
| S6H | 30 | 30,000 | 5.26 | 3.55 | 7.51 |
| P7B | 30 | 30,000 | 5.26 | 3.55 | 7.51 |
| J8Y | 25 | 25,000 | 5.26 | 3.41 | 7.77 |
| K9V | 25 | 25,000 | 5.26 | 3.41 | 7.77 |
| G1S | 25 | 25,000 | 5.26 | 3.41 | 7.77 |
| N7L | 25 | 25,000 | 5.26 | 3.41 | 7.77 |
| H7G | 20 | 20,000 | 5.26 | 3.21 | 8.13 |
| J6K | 20 | 20,000 | 5.26 | 3.21 | 8.13 |
| A0H | 20 | 20,000 | 5.26 | 3.21 | 8.13 |
| G0J | 35 | 36,000 | 5.12 | 3.56 | 7.12 |
| K1J | 25 | 26,000 | 5.06 | 3.28 | 7.47 |
| H8N | 25 | 26,000 | 5.06 | 3.28 | 7.47 |
| G0R | 80 | 84000 | 5.01 | 3.97 | 6.24 |
| H4V | 20 | 21,000 | 5.01 | 3.06 | 7.74 |
| J0C | 20 | 21,000 | 5.01 | 3.06 | 7.74 |
| K7L | 20 | 21,000 | 5.01 | 3.06 | 7.74 |
| M6L | 20 | 21,000 | 5.01 | 3.06 | 7.74 |
| L2E | 20 | 21,000 | 5.01 | 3.06 | 7.74 |
| R0M | 20 | 21,000 | 5.01 | 3.06 | 7.74 |
| G0A | 90 | 95,000 | 4.99 | 4.01 | 6.13 |
| H7W | 35 | 37,000 | 4.98 | 3.47 | 6.92 |
| T2E | 30 | 32,000 | 4.93 | 3.33 | 7.04 |
| G7X | 25 | 27,000 | 4.87 | 3.15 | 7.19 |
| K2C | 25 | 27,000 | 4.87 | 3.15 | 7.19 |
| B3M | 25 | 27,000 | 4.87 | 3.15 | 7.19 |
| L0N | 25 | 27,000 | 4.87 | 3.15 | 7.19 |
| J4B | 35 | 38,000 | 4.85 | 3.38 | 6.74 |
| T2J | 45 | 49,000 | 4.83 | 3.53 | 6.47 |
| B0P | 40 | 44,000 | 4.78 | 3.42 | 6.52 |
| M9R | 30 | 33,000 | 4.78 | 3.23 | 6.83 |
| J0J | 35 | 39,000 | 4.72 | 3.29 | 6.57 |
| J3G | 25 | 28,000 | 4.70 | 3.04 | 6.94 |
| J2S | 25 | 28,000 | 4.70 | 3.04 | 6.94 |
| J2B | 25 | 28,000 | 4.70 | 3.04 | 6.94 |
| J9X | 25 | 28,000 | 4.70 | 3.04 | 6.94 |
| G1C | 30 | 34,000 | 4.64 | 3.13 | 6.63 |
| J6A | 30 | 34,000 | 4.64 | 3.13 | 6.63 |
| J0P | 30 | 34,000 | 4.64 | 3.13 | 6.63 |
| J0Y | 30 | 34,000 | 4.64 | 3.13 | 6.63 |
| L9W | 30 | 34,000 | 4.64 | 3.13 | 6.63 |
| G0X | 55 | 63,000 | 4.59 | 3.46 | 5.98 |
| N0L | 40 | 46,000 | 4.58 | 3.27 | 6.23 |
| K0M | 40 | 46,000 | 4.58 | 3.27 | 6.23 |
| J0L | 65 | 77,000 | 4.44 | 3.43 | 5.66 |
| A0A | 45 | 54,000 | 4.39 | 3.20 | 5.87 |
| J0H | 45 | 54,000 | 4.39 | 3.20 | 5.87 |
| B0J | 40 | 48,000 | 4.39 | 3.13 | 5.97 |
| J0R | 40 | 49,000 | 4.30 | 3.07 | 5.85 |
| N0P | 45 | 56,000 | 4.23 | 3.08 | 5.66 |
| S0G | 50 | 63,000 | 4.18 | 3.10 | 5.51 |
| N0M | 50 | 64,000 | 4.11 | 3.05 | 5.42 |
| J0K | 100 | 132,000 | 3.99 | 3.24 | 4.85 |
| K0K | 75 | 103,000 | 3.83 | 3.01 | 4.80 |

| FSAs with zero incidence | Raw Incidence | Reference Population | Incidence per 100,000 | Adjusted Lower IC (9%%) | Adjusted upper IC (95%) |
| --- | --- | --- | --- | --- | --- |
| T6T | 0 | 12,000 | 0 | 0.000 | 16.088 |
| V0T | 0 | 10,000 | 0 | 0.000 | 19.306 |
| V2L | 0 | 10,000 | 0 | 0.000 | 19.306 |
| V2N | 0 | 32,000 | 0 | 0.000 | 6.033 |
| V3X | 0 | 17,000 | 0 | 0.000 | 11.356 |
| V3Y | 0 | 16,000 | 0 | 0.000 | 12.066 |
| V5T | 0 | 21,000 | 0 | 0.000 | 9.193 |
| V5V | 0 | 23,000 | 0 | 0.000 | 8.394 |
| V8C | 0 | 10,000 | 0 | 0.000 | 19.306 |
| X0A | 0 | 15,000 | 0 | 0.000 | 12.871 |
